# The Action of Chloroform and Ether

**Published:** 1891-02

**Authors:** 


### The Action of Chloroform and Ether.

Professor MacWilliam, of Aberdeen, has just published the results of a long and elaborate investigation of the above subject, covering a period of two years, in which a new method of studying the heart and blood-vessels has been adopted. His report is presented to the Scientific Grants Committee of the British Medical Association. It is demonstrated that chloroform exerts a direct influence on the heart—depressing its energy, diminishing its tone, and dilating its chambers. Moreover, such a depressing effect may be brought about by chloroform when given mixed with abundance of air (under four per cent. of chloroform vapor), and when the amount of the anæsthetic administered is not sufficient to abolish the conjunctival reflex. The mode of cardiac failure under chloroform is not a sudden arrest of the rhythmic action, but a more or less sudden dilatation and enfeeblement of the organ, causing the rhythmic contraction to be ineffective. Examples are given of death from cardiac failure while the respiration went on for many minutes. The different action of ether as compared with chloroform is strikingly marked. Ether usually causes abolition of the conjunctival reflex and profound anæsthesia with no depression of the heart, or only a brief and trivial one.

